# Chiral Phase Transfer Catalysis in the Asymmetric Synthesis of a 3,3-Disubstituted Isoindolinone and Determination of Its Absolute Configuration by VCD Spectroscopy

**DOI:** 10.3390/molecules25102272

**Published:** 2020-05-12

**Authors:** Guglielmo Monaco, Maximilian Tiffner, Antonia Di Mola, Wouter Herrebout, Mario Waser, Antonio Massa

**Affiliations:** 1 Dipartimento di Chimica e Biologia, Università di Salerno, Via Giovanni Paolo II, 132, 84084 Fisciano (SA), Italy; toniadimola@libero.it; 2 Institute of Organic Chemistry, Johannes Kepler University Linz, Altenbergerstr. 69, 4040 Linz, Austria; maximilian.tiffner@gmail.com (M.T.); mario.waser@jku.at (M.W.); 3 Department of Chemistry, University of Antwerp, B-2020 Antwerp, Belgium; wouter.herrebout@uantwerpen.be

**Keywords:** organocatalysis, phase transfer catalysis, vibrational circular dichroism, heterocycles, isoindolinones

## Abstract

In this work we report our endeavors toward the development of an asymmetric synthesis of a 3,3-disubstituted isoindolinone, dimethyl 2-(1-methyl-3-oxoisoindolin-1-yl)malonate, via asymmetric cascade reaction of 2-acetylbenzonitrile with dimethylmalonate and the determination of its absolute configuration (AC) by vibrational circular dichroism (VCD). Bifunctional ammonium salts, derived from *trans-*1,2-cyclohexanediamine in combination with inorganic bases under phase transfer conditions, were the most effective catalytic systems, leading to the target in high yields and moderate enantioselectivity. An efficient process of heterochiral crystallization allowed the increase of the enantiopurity up to 96% ee and in an acceptable overall yield. An important aim of the present work is the comparison of different VCD methodologies for AC determination of the target compound.

## 1. Introduction

**Figure 1:**
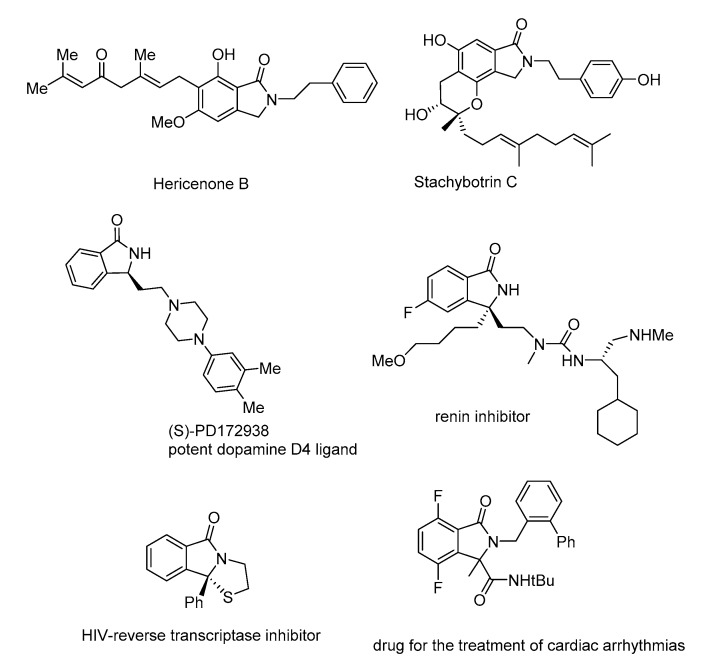
Hericenone B and stachybotrin C are two selected examples of 3-unsubstituted isoindolinones from natural sources; (*trans*)-PD172938 is a representative known bioactive 3-mono-substituted derivative; the last 3 compounds represent almost all the known bioactive 3,3-disubstituted isoindolinones.

Isoindolinones are privileged heterocycles with a wide range of biological activities and applications (Figure 1) [1,2,3]. These compounds are characterized by a γ-lactam fused with a benzene ring, which can be either unsubstituted, mono- or di-substituted in 3 position (Figure 1). Despite the great number of articles describing synthesis and properties of unsubstituted or 3-mono substituted isoindolinones, asymmetric synthesis of 3,3-disubstituted derivatives has received relatively little attention [2,3,4,5,6,7,8,9,10], because of the well-known difficulties in the asymmetric construction of quaternary stereocenters [11]. In the course of our recent research activity, we have developed a straightforward approach to racemic 3,3-disubstituted isoindolinones via K_2_CO_3_ catalyzed cascade reactions of 2-acylbenzonitriles in the presence of a range of nucleophiles [12].

Promising enantioselectivities have been obtained using nitromethane as pro-nucleophile in a nitro-aldol initiated cascade type reaction in the presence of bifunctional ammonium salts derived from *trans*-1,2-cyclohexanediamine [13]. Considering the importance to develop new asymmetric one-pot processes for atom- and step-economy [14], we were interested to investigate further asymmetric versions of such a straightforward strategy to access quaternary stereocenter-containing isoindolinones by using different nucleophiles. The use of malonate diesters is of great interest because the introduction of such a group on the side chain of the isoindolinone ring could allow further useful transformations [15]. On the basis of these considerations we have investigated asymmetric cascade reactions of 2-acetylbenzonitrile with dimethyl malonate to obtain enantioenriched dimethyl 2-(1-methyl-3-oxoisoindolin-1-yl)malonate **1**. The absolute configuration (AC) of **1** is of interest in view of its future use for the synthesis of chiral substances of pharmacological interest. Vibrational circular dichroism (VCD) is a powerful method for that task [16,17] and we are developing new statistical tools [18,19] to cope with unavoidable limitations in the DFT calculations, like basis set size, functional choice, description of solvent and anharmonicities. These limitations are especially relevant for species with more than a single stereocenter or with many thermally accessible conformations [20,21], like the present compound. For similar situations extension to higher level of calculation is generally computationally very demanding. Rather than resorting to DFT calculations, simpler approaches can also be tried considering the presence of three relatively close carbonyl groups. Indeed, some years ago we have successfully assigned the absolute configuration of the analogous compound dimethyl 2-(3-oxoisoindolin-1-yl) malonate, studying only the carbonyl region of the VCD spectrum and adopting the semiempirical extended coupled oscillator (ECO) model [22], coupled with force field calculations [23]. An even simpler approach, considering only the sign of the O=C---C=O dihedral angle, has been applied successfully to many compounds by Taniguchi and Monde [24]. The latter approach has found considerable criticism in literature [25,26]. As a contribution to understand scope and limitations of the simplified approaches, in this paper, beyond assignment of AC based on quantum chemical calculations, we are going to investigate the applicability of the simplified approaches in case of **1**.

## 2. Results and Discussion

### 2.1. Screening of Different Organocatalytic Systems

**Scheme 1:**
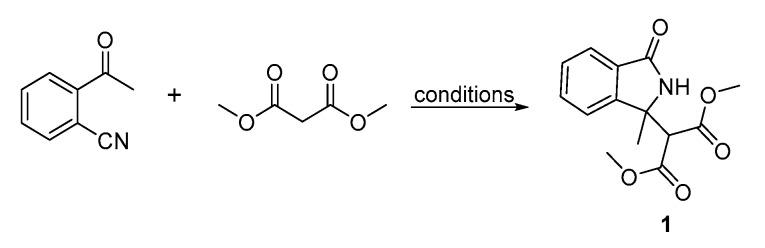
Cascade reaction of 2-acetylbenzonitrile.

**Figure 2:**
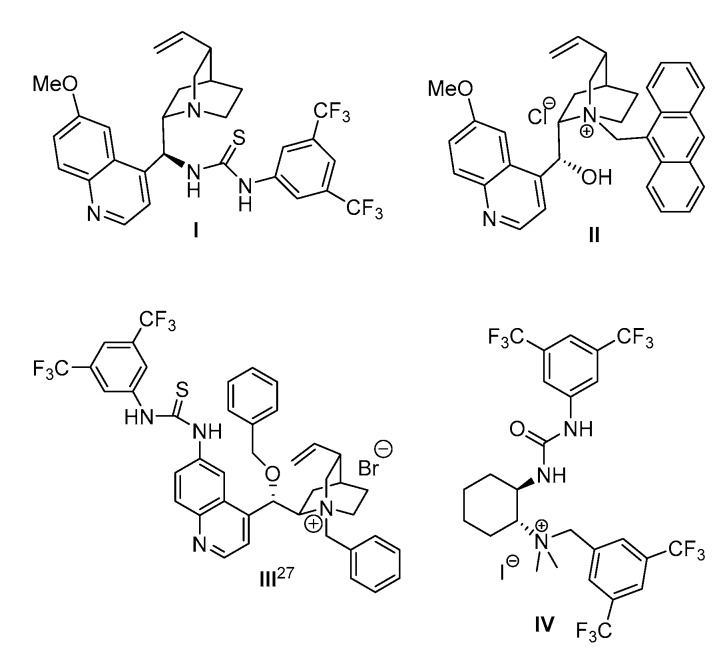
Selected chiral tertiary amines and ammonium salts tested herein.

Searching a suitable chiral organocatalyst for the reaction between 2-acetylbenzonitrile and dimethyl malonate (Scheme 1) among four recently developed systems [27], we soon realized that bifunctional chiral ammonium salts as **IV** in the presence of inorganic bases under phase transfer conditions were more effective than other common PTCs or chiral tertiary amines (Figure 2 and Table 1). Albeit relatively moderate in enantioselectivity for this transformation (Entry 5), this class of bifunctional ammonium salts derived from *trans*-1,2-cyclohexanediamine can be considered privileged catalysts in asymmetric cascade reactions of 2-acetylbenzonitrile and 2-formylbenzonitriles [13,15].

With the aim to improve the enantioselectivity of the reaction further, a systematic screening of the reaction conditions with catalyst **IV** was performed next (Table 2). Among different solvent-base combinations the best results were obtained using 3 eq. of K_3_PO_4_ in DCM using 5 mol% of the catalyst, while non-halogenated solvents were found to be less promising while carbonate bases (i.e., K_2_CO_3_) turned out to be suited as well (Table 2).

**Figure 3:**
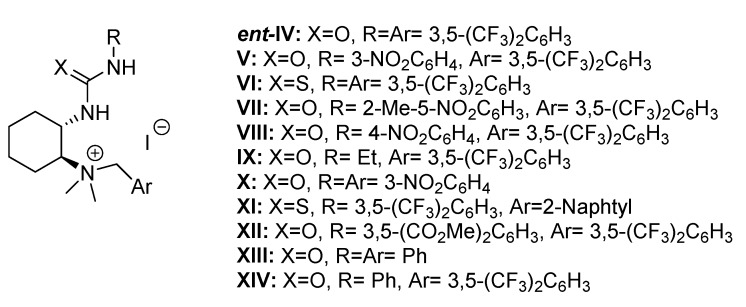
Bifunctional ammonium salts derived from *trans*-(*S*,*S*)-1,2-cyclohexanediamine.

A large screening of chiral 1,2-diaminocyclohexane catalysts, *ent-***IV**–**XIV**, was carried out next. In all these cases, the catalysts were derived from the (*S*,*S*)-1,2-diaminocyclohexane and, as one could expect, afforded the opposite enantiomer. This systematic testing highlighted that, as a general feature, the presence of strong electron-withdrawing groups like CF_3_ usually lead to higher enantioselectivities (up to 50% ee) and higher yields in shorter reaction times even in the presence of only 5 mol% of the catalyst (Figure 3 and Table 3). The urea derivatives were found to be better suited than the analogous thioureas, leading to higher yields (Entry 1 vs. Entry 3). The decrease of the reaction temperature at −20°C led to lower enantioselectivity, while higher base amount and longer reaction times were necessary to attain comparable yields (Entry 12). Under the best conditions of entry 1 of Table 3, the reaction was also scaled up to 100 mg of 2-acetylbenzonitrile, using catalyst **IV** (with *R*,*R* configuration), obtaining similar results in terms of yield and ee (90% and 50% respectively). The enantiopurity of the product was further improved by means of a heterochiral crystallization process (**1** crystallizes as racemate), leading to the isolation of **1** from the mother liquor in up to 96% ee and in an acceptable efficiency (45% yield), thus resulting in an overall process (asymmetric catalytic cyclization followed by crystallization) allowing for considerable quantities of almost enantiopure isoindolinone **1** from simple starting materials. Since the enantioenriched **1** is an amorphous wax, X-ray crystallography could not be applied in the determination of the AC. This issue prompted us to reinvestigate several methods available to assign AC via VCD spectroscopy.

**Scheme 2:**
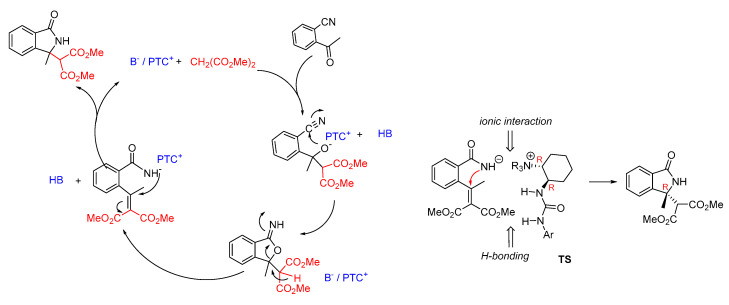
Proposed mechanistic pathway and transition state.

On the basis of previous reports on the reactions of 2-formylbenzonitriles [15] and 2-acylbenzonitriles [2,12,13], a tandem mechanism can be proposed according to Scheme 2. The presence of the cyano group in 2-position of the aromatic ring has the advantage to drive the unfavorable addition reaction toward the formation of the cyclic imidate. The presence of an acidic proton at the α position on the side chain of the imidate permits a further Dimroth type rearrangement via deprotonation, ring opening and intramolecular aza-Michael conjugated addition leading to the title 3,3-disubstituted isoindolinone. The catalytic system formed by the ionic couple B^−^/PTC^+^ mediates all the steps. The base is involved in the deprotonation of dimethylmalonate and of the imidate intermediate, while the chiral ammonium salt probably will form an ionic couple with the anionic amide in the stereochemical determining step, the intramolecular aza-Michael reaction. The catalyst is supposed to interact with the substrate in the intramolecular aza-Michael type cyclization via chiral ion pair formation. Here several activation modes may be possible like ion pair formation between the amide anion and the ammonium group of the catalyst with simultaneous H-bonding interaction of the urea with the ester group or the inverse activation mode (as exemplified recently for a different application with these catalysts [28]). However the nature of this exact interaction mode remains speculative, but it is obvious that the bifunctional nature of the catalyst is crucial for obtaining the promising enantioselectivities achieved herein (the *R*,*R*-catalyst gives the *R* product; configuration determined as described in the following chapter).

### 2.2. Determination of the Absolute Configuration by VCD

#### 2.2.1. The Experimental Spectra

**Figure 4:**
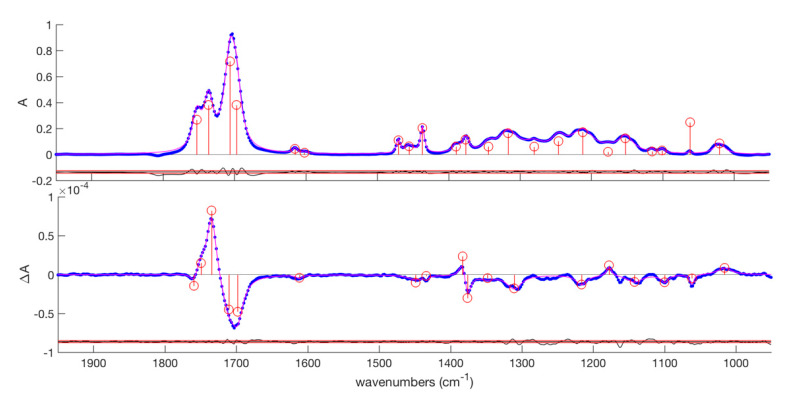
Vibrational absorption (VA) and vibrational circular dichroism (VCD) infrared spectra of (+)-**1**: the experimental trace is dotted blue, the hardly visible Lorentzian fit is magenta, central frequencies of Lorentzian peaks are marked with circle-ended stems. The traces below the spectra are the residuals flanked by an indication of standard error. The small negative peak in the VA spectrum at 1808 cm^−1^ is likely due to a trace amount of phosgene formed by oxidation of the non-stabilized chloroform used to dissolve the racemic sample [29].

The infrared radiation has been used to record VA (Vibrational Absorption) and VCD (Vibrational Circular Dichroism) spectra of (+)-**1**, shown in Figure 4. The carbonyl region of both VA and VCD spectra shows three peaks in both VA and VCD spectra. In order to estimate the error on the spectra, which is a request of the model-averaging method, the spectra have been fitted with sum of Lorentzians. The 22 central frequencies coming from the fit of the VA spectrum have been used to build a guess of the VCD spectrum. The VCD fit was then pruned of peaks of too law intensity and enriched with two peaks that were not clearly visible in the VA spectrum, ending with a fit with 18 Lorentzian peaks. The fits allowed to estimate experimental errors $\sigma_{exp}^{VA}$ = 1.0 × 10^−2^ and $\sigma_{exp}^{VCD}$ = 1.6 × 10^−6^. The model as sum of Lorentzians is hardly distinguishable from the experimental data. Notably, 4 and 5 Lorentzians have been used to model the carbonyl region for the VA and VCD spectrum, respectively. The presence of more than three normal modes in the carbonyl region is an experimental indication of more than one thermally populated conformer for **1**, which has only three carbonyl groups. The most significant features of the spectra are the carbonyl peaks; in the VCD spectrum a strong negative couplet (1735 and 1703 cm^−1^) is observed.

#### 2.2.2. Conformational Analysis

**Figure 5:**
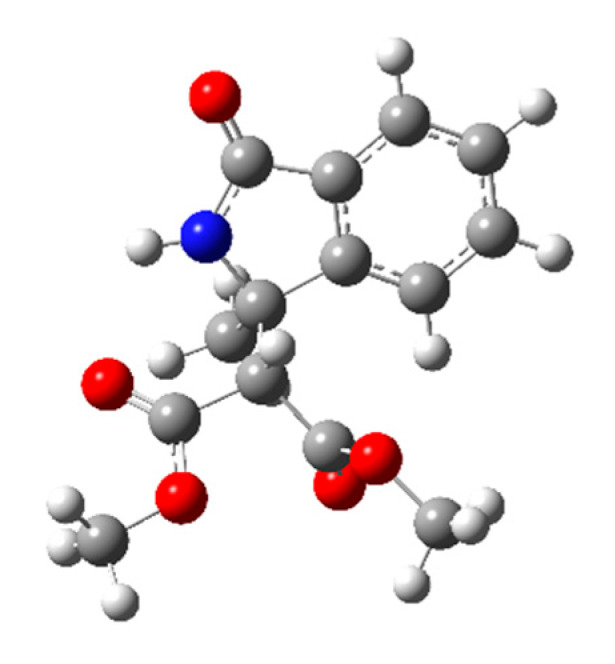
Lowest energy conformer of **1**, disclosing an H-bond between the N-H and the carbonyl.

The conformational analysis indicate the presence of 4 conformers with H-bond between the N-H and the carbonyl, 6 higher energy conformers with H-bond between the N-H and the ester oxygen, and 4 more conformers devoid of H-bond. Two of the latter have energies comparable with those of conformers with H-bond to ester oxygen. The lowest energy conformer is shown in Figure 5. Relevant geometrical information for unique conformers are gathered in Table S1.

#### 2.2.3. AC Assignment through Model-Averaged DFT Calculations

**Figure 6:**
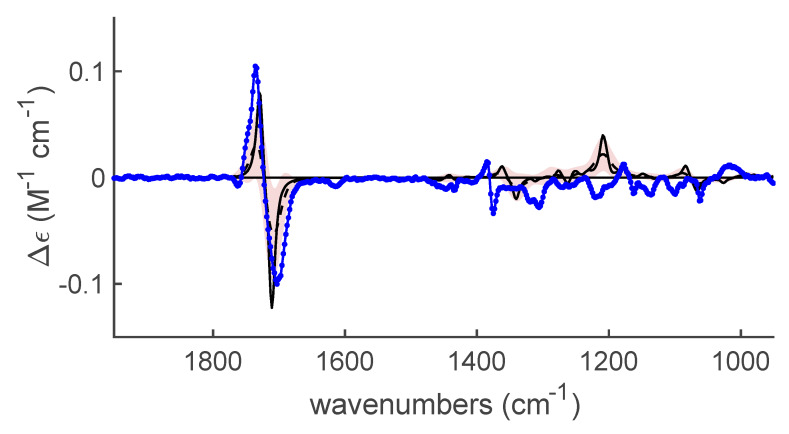
Experimental VCD spectrum of (+)-**1** (blue) and the MA-PCM-B3LYP/6-31G* spectrum of (*R*)-**1** (dashed line) coming with an error estimate (shaded area). The continuous black line is the plain PCM-B3LYP/6-31G* spectrum. Computed frequencies have been scaled by a factor λ = 0.948, determined by least-squared fitting.

In the model-averaging (MA) approach, the VCD spectrum is modeled using eight levels of calculation (B3LYP/TZ2P [30], B3LYP/cc-PVTZ [30,31,32], B3LYP/6-31G* [21], B3PW91/TZ2P [30], B3PW91/cc-PVTZ [30,31], B97D/TZ2P [23], ω-B97XD/6-31G* [21], ω-B97XD/6-311++G** [33]), adopted in previous successful AC assignment of small organic molecules, using in all cases the PCM method [34], to model the effect of the solvent. DFT computations are used to obtain an average spectrum, which is accompanied by an estimate of error bounds. Rather than averaging the computed spectra directly, the computations are used to estimate standard deviations on spectral parameters needed to model the spectrum. Final standard deviations proposed on central frequency, electric and magnetic dipole moment, *μ* and *m*, and the angle *ξ* formed by them were σ($\tilde{\nu }$_0_) = 10 cm^−1^, σ(*μ*) = 2 × 10^−20^ esu cm, σ(*m*) = 1.5 × 10^−24^ esu cm, σ(*ξ*) = 10°. Standard deviations of energies, enthalpies and free enthalpies were 0.05, 0.10 and 0.5 kcal mol^−1^. These values are likely underestimates in case of a H-bonded system, like **1**, but, considering the following results, we have not considered re-parametrization. The uncertainties in the modeled spectra can then be summed in quadrature with the experimental errors to have a total variance of the difference of the experimental spectrum and the model spectrum: $\sigma_{i}^{2}=\sigma_{i,exp}^{2}+\sigma_{i,MA-M}^{2}$ Dividing each point of measured and experimental spectrum by the total standard deviation one gets standardized residuals and several goodness-of-fit indicators (GOFIs) can be used to select the best AC. In addition to the commonly employed root-mean-square error (RMSE), which is heavily affected by outliers, more robust indicators like the cosine similarity (COSI) [20,35] and the mean absolute error over the mean of the absolute spectrum ratio (MMAR) [19,36] can be used. It is important to note that errors on these indicators can be obtained by the bootstrap method [37,38], so that the preference of one AC over a competing one is corroborated by a statistical error bound. While comparing two stereochemical models, the worst will lead to higher RMSE, higher MMAR and lower COSI. Rather than using all the eight computations, on the assumption that the main effect of MA comes from the widened variances and that one chooses a method which is not very far from the averaged spectrum, we have been computing a MA-PCM-B3LYP/6-31G* and obtained results as effective as the whole MA approach [19]. This simplified approach gives the MA spectrum shown in Figure 6.

The agreement of experimental and MA spectrum in the carbonyl region is very good, but it is poor in the remaining part of the spectrum. The poor performance is probably not surprising considering that the basis 6-31G*, devoid of polarization and diffuse function on hydrogen, is under-dimensioned for the description of H-bonded systems. Moreover, the least-squares determination of the frequency scaling factor is largely determined by the strong carbonyl region, and it can be realized by inspection of the spectrum that a smaller value for the scaling factor can lead to better agreement in the fingerprint region. At any rate, notwithstanding this poor performance off the carbonyl region, we note that the GOFIs and their errors clearly point to the (*R*) configuration (Table 4). Only the COSI is able to select the (*R*) configuration without model-averaging, while all three GOFIs concur in selecting the (*R*) configuration, using model-averaging.

#### 2.2.4. AC Assignment through the ECO Model

**Figure 7:**
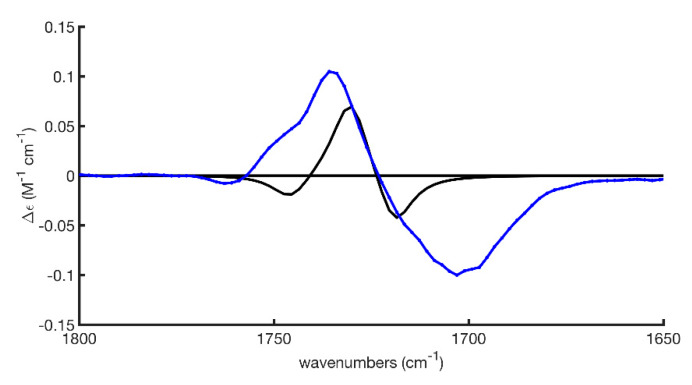
The extended coupled oscillator (ECO) VCD spectrum (black) superposed to the experimental VCD spectrum.

Having elucidated the AC of **1**, we were then investigating the performance of simplified approaches, starting with the ECO [21]. The modeled VCD of (*R*)-**1** in the carbonyl region is shown in Figure 7, and it is in nice qualitative agreement with the experiment: all three signs of the experimental pattern are correctly predicted.

The fact that two of the three dichroic signals are more intense suggest that two of the carbonyls are more strongly coupled than the third one. Indeed, the carbonyl groups of the malonate are closer to each other and the coupling energies as computed by the ECO method always indicate that they have a higher value as compared with the couplings with the amidic CO group. As a consequence, it should be possible to identify a positive or negative couplet (−/+ or +/− dichroic signals going from high to low frequency) for each conformer. Notably the two lowest energy conformers happen to have a negative couplet. Considering that the ECO spectrum is the result of a Boltzmann averaging and that we have kept the same population of conformers coming from the DFT calculations, the agreement between ECO and DFT indicates that the thermally populated conformers have a dichroic pattern similar to the DFT calculations. As can be seen from Table S2, this occurs for the two lowest energy conformers, and overall 11 times out of 14. In the 3 cases where a significant difference in the designation of the couplet is observed, the conformers have H-bonding, which is a feature not considered by the ECO model.

#### 2.2.5. Applicability of Taniguchi and Monde Approach

Taniguchi and Monde have proposed that the sign of C=O couplets can be predicted just considering the dihedral angle O=C-C=O of the most stable conformer, and they have demonstrated the reliability/validity of their model by experimental means in several cases [24]. Their assumption has been discussed in detail within the coupled-oscillator model [25,26]. Beyond warnings about the necessity of (1) having the two C=O being almost isolated from other vibrations (2) facing conservative couplets, and (3) having the C=O not coplanar, a major concern comes from the relative position of the in-phase and the out-of-phase vibrations. Borrowing the notation of Reference 25, the in-phase (+ sign) and out-of-phase (−sign) vibrations of the C=O groups have absorption frequency and rotational strength given by:

*ν*_±_ = *ν*_0_ ± (*hc*)*^−^*^1^*μ^2^ d^−3^* [sin *α*_1_ sin *α*_2_ cos *φ* + 2cos*α*_1_ cos*α*_2_] (1)

**Figure 8:**
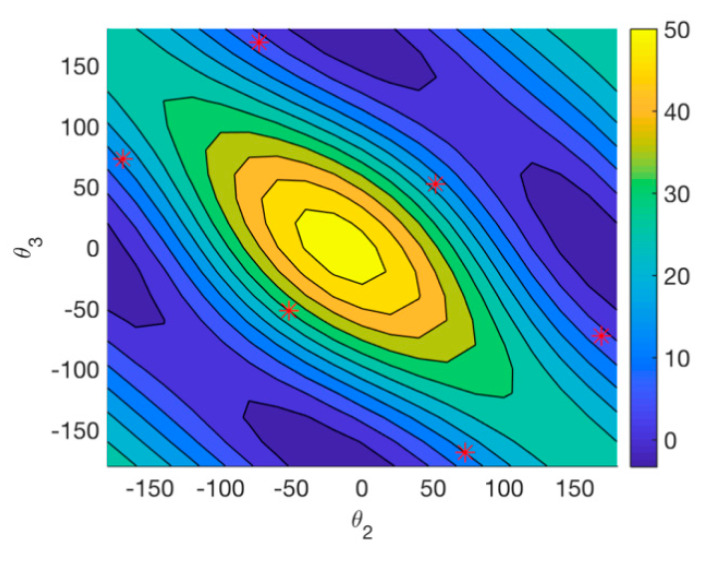
Scan of Δ*ν* as a function of the two central torsion angles of dimethylmalonate. The red asterisks mark the occurrence of almost degenerate minima at the B3LYP/6-31G* level.

*R*_±_ = ∓*πν*_±_/2 *d μ*^2^ sin *α*_1_ sin *α*_2_ sin*φ* (2)

where *d* is the distance of the two C of the C=O groups, *α_1_* and *α_2_* are the angles of the C=O bonds with the C-C connecting line, and *φ* is the dihedral angle O=C-C=O, *μ* is the transition dipole moment, and *h* and *c* have their usual meaning. Considering that sin*α*_1_ and sin*α*_2_ are always positive, Equation (2) states that the out-of-phase vibration has the same sign of the dihedral angle *φ.* However, in order for the *couplet* to have the same sign of *φ* it should also be verified that the out-of-phase vibration occurs at lower energy, i.e., Δ*ν* = *ν_+_* − *ν_−_* > 0. Interestingly, for all 14 conformers the sign of the couplet is the same of the sign of the dihedral *φ*, which points to Δ*ν* > 0. This fact is mostly a geometrical result. For two 1,2 carbonyl groups with a valence angle of 90°, one expects that half of the conformational space will be characterized by a repulsive interaction of the carbonyl groups and then by Δ*ν* < 0. This prediction should be corrected for a valence angle different from 90° and by the intervention of more than 1 bond between the carbonyl groups, as happens for the present case of 1,3-dicarbonyl. As a representative model of our case, we considered dimethylmalonate and scanned Δ*ν* as a function of the two dihedral angles CC(=O)CC(=O), θ_2_ and θ_3_. As can be seen from Figure 8, only a small fraction of conformational space has Δ*ν* < 0 and that zone is far from the minimum energy values (Figure 8). These results suggest that in the case of 1,3-dicarbonyl compounds and in absence of H-bond formation, the simple Taniguchi and Monde should well work in the majority of cases.

## 3. Materials and Methods

### 3.1. General Remarks

Reactions were performed using commercially available compounds without further purification and analytical grade solvents. 2-acetylbenzonitrile was prepared according to reported procedures [12]. All the reactions were monitored by thin layer chromatography (TLC) on precoated silica gel plates (0.25 mm) and visualized by fluorescence quenching at 254 nm. Flash chromatography was carried out using silica gel 60 (70–230 mesh, Merck, Darmstadt, Germany). The ^1^H-NMR spectra were recorded on Bruker DRX 300 spectrometer (300 MHz, ^1^H, Bruker, Rheinstetten, Germany). Spectra were referenced to residual CHCl_3_ (7.26 ppm, ^1^H).

### 3.2. Procedure of Asymmetric Synthesis and Crystallization of ***1***

In a typical procedure, a mixture of 2-acetylbenzonitrile (14 mg, 0.1 mmol) in CH_2_Cl_2_ (1.8 mL), catalyst IV (5 mol%), anhydrous K_3_PO_4_ (64 mg, 3 eq.) and dimethylmalonate (34 μL, 3 eq.) was stirred at room temperature till disappearance of the starting material (TLC, Hexane/Ethyl acetate, 4:6). The solution was filtered and purified on silica gel (Hexane/Ethyl acetate, 70:30 to 50:50) obtaining a white solid. Yield: 85% (23.5 mg, 0.085 mmol). Spectroscopic data in accordance with literature [12]. Ee: 50%, Chiralpak IA3, Hex/IPA 80:20, 0.6 mL/min, λ: 254 nm, t: 17.52 min and 20.90 min. The reaction was also scaled up to 100 mg of 2-acetylbenzonitrile with cat. IV (*R*,*R*) (0.714 mmol) giving similar results in terms of yield and ee.

Procedure for crystallization of **1a**. A sample of 40 mg, obtained from the above scale-up procedure, was dissolved in a mixture of CHCl_3_ (500 µL) and hexane (1.00 mL) at room temperature and then left at −20 °C for 72 h. The enantio-enriched product was recovered by filtration and evaporation of the solution, giving 18 mg with 96% ee. ${\left[\alpha \right]}_{D}^{20}$: +104.0 (c 0.80, CHCl_3_).

### 3.3. Determination of the Absolute Configuration by VCD

Vibrational absorption (VA) and VCD spectra were recorded on a BioTools dual-PEM ChiralIR-2X spectrometer (BioTools, Jupiter, FL, USA) at room temperature. The PEMs were optimized for 1400 cm^−1^, and a resolution of 4 cm^−1^ was used throughout. For measurements on 2, solutions were prepared by dissolving 3.8 mg in 100 μL of CDCl_3_. All spectra were recorded using BaF_2_ windows and a spacer of 50 μm. The solution spectra were averaged over 100,000 scans, whose acquisition lasted 36 h. Baseline corrections were introduced by subtracting the spectra of racemate and solvent.

### 3.4. Computations

Ab initio calculations have been performed by Gaussian 09 [39]. Conformers of **1** have been first guessed with Confab [40], and then optimized at the B3LYP/6-31G* level using the PCM method [34] to model the effect of chloroform. Model-averaged spectra have been generated using the relative energies reported in the Supporting Information at the temperature of 298 K.

## 4. Conclusions

Dimethyl 2-(1-methyl-3-oxoisoindolin-1-yl)malonate **1** has been synthesized in highly enantioenriched form. This has given us the occasion to reinvestigate several methods available to assign its AC via VCD spectroscopy for this rather flexible molecule. We have first used the model-averaging method, which quantifies the unavoidable limitations of the DFT calculations, using error bound for the predicted spectra, as well as robust goodness-of-fit indicators [18,19]. This method allows a confident assignment of (+)-**1** to (*R*)-**1**, even using a low level of computation, such as PCM-B3LYP/6-31G*. As the assignment substantially comes from the carbonyl region, the simplified the extended-coupled oscillator (ECO) method [22,23] has also been considered. This method also leads to the same assignment. Eventually, the method of Taniguchi and Monde [24], considering only the torsion angle formed by two strongly coupled C=O groups in the lowest energy conformer, also yields to the same conclusion. Detailed analysis shows that a confident use of the simplified ECO can be hampered by the formation of H-bonds. As for the Taniguchi and Monde model, whose limitations in case of reversal of symmetric and antisymmetric C=O stretching bands have been clearly highlighted [26], it has been shown that it turns out effective in almost all cases where the ECO works, in case of 1,3-dicarbonyl, mainly for geometric reasons.

## Figures and Tables

**Table 1:** Preliminary screening of chiral tertiary amines and ammonium salts **I**–**IV**.

Entry	Base (1 eq.)	Catalyst	Time (d)	Yield (%) ^a^	ee (%) ^b^
1	None	**I**	7 d	No react.	--
2	K_2_CO_3_	**II**	7 d	78	10
3	KOH	**II**	7 d	41	Rac
4	K_2_CO_3_	**III**	3 d	53	Rac
5	K_2_CO_3_	**IV**	7 d	90	48

^a^ Isolated yields. ^b^ Determined by chromatography on chiral stationary phase.

**Table 2:** Optimization of reaction conditions with catalyst **IV**.

Entry	IV (mol%)	Base (eq)	Solvent	Time (h)	Yield (%) ^a^	ee (%) ^b^
1	10	K_2_CO_3_ (1)	DCM	60h	11	50
2	10	K_2_CO_3_ (3)	DCM	60h	83	50
3	10	K_2_CO_3_ (aq.50%)	DCM	60h	9	50
4	10	Cs_2_CO_3_ (1)	DCM	20	95	28
5	10	K_3_PO_4_ (1)	DCM	44	80	50
6	5	K_3_PO_4_ (3)	DCM	20	85	50
7	5	K_2_HPO_4_ (3)	DCM	20	No react	--
8	5	KH_2_PO_4_ (3)	DCM	20	No react	--
9	5	K_3_PO_4_ (3)	toluene	20	66	22
10	5	K_3_PO_4_ (3)	MtBE	20	78	34
11	5	K_3_PO_4_ (3)	DCE	20	75	50
12 ^c^	5	K_3_PO_4_ (5)	DCM	48	75	30

^a^ Isolated yields. ^b^ Determined by chromatography on chiral stationary phase. ^c^ Reaction performed at −20 °C.

**Table 3:** Catalyst screening ^a^.

Entry	Catalyst	Time (h)	Yield (%) ^b^	ee (%) ^c^
1	*ent*-**IV**	20	85	50
2	**V**	72	83	48
3	**VI**	72	20	50
4	**VII**	20	85	46
5	**VIII**	72	82	52
6	**IX**	72	18	22
7	**X**	72	18	44
8	**XI**	72	19	26
9	**XII**	20	89	26
10	**XIII**	72	42	7
11	**XIV**	72	74	49

^a^ A typical reaction was carried out on 0.1 mmol of 2-acetylbenzonitrile in CH_2_Cl_2_ (1.8 mL), with catalyst *ent-*IV (5 mol%), anhydrous K_3_PO_4_ (3 eq.) and dimethylmalonate (3 eq.). ^b^ Isolated yields. ^c^ Determined by chromatography on chiral stationary phase.

**Table 4:** Bootstrap values of means, <·>*_b_*, and standard deviations, *sb* (<·>*_b_*), of three goodness-of-fit indicators (GOFIs) for the two possible AC of **1**, computed either by a plain PCM-B3LYP/6-31G* calculation or by its MA version. The last two columns of the table report the differences of the GOFIs computed for the (*S*) and (*R*) configuration, and an estimate of the error on the difference computed by standard error propagation: *s*^2^ (Δ<·>) = *s*^2^ (<·>*_R_*) + *s*^2^ (<·>*_S_*).

	(*S*)		(*R*)		(*S*)–(*R*)	
Plain DFT	<∙>_b_	*s*(<∙>_b_)	<∙>_b_	*s*(<∙>_b_)	∆<∙>_b_	*s*(∆<∙>_b_)
RMSE	0.0190	0.0017	0.0190	0.0017	0.000_04_	0.002_43_
MMAR	1477	9	1475	8	_2_	1_2_
COSI	−0.9979	0.0004	0.9980	0.0004	−1.9959	0.0005
MA-DFT	<∙>_b_	*s*(<∙>_b_)	<∙>_b_	*s*(<∙>_b_)	∆<∙>_b_	*s*(∆<∙>_b_)
RMSE	2.21	0.10	2.01	0.11	0.2_1_	0.1_4_
MMAR	1.08	0.01	0.99	0.01	0.09_7_	0.01_9_
COSI	−0.15	0.05	0.15	0.05	−0.31	0.07

## Supplementary Materials

The following are available online. Table S1: Geometrical parameters of the conformers of **1**. Table S2: Rotational strengths for the three carbonyl resonances obtained for the 14 conformers of **1**. Figure S1: Atomic numbering used to define the geometrical parameters of the conformers of **1**. HPLC trace for *rac*-**1** prepared according to ref. 12. HPLC trace for enantioenriched *R*-**1** (experiment coming from scale-up). HPLC trace for enantioenriched *R*-**1** after crystallization.
