# Correction: Monaco et al. Chiral  Phase Transfer Catalysis in the Asymmetric Synthesis of a 3,3-Disubstituted Isoindolinone and Determination of Its Absolute Configuration by VCD Spectroscopy. *Molecules* 2020, *25*, 2272

**DOI:** 10.3390/molecules28114272

**Published:** 2023-05-23

**Authors:** Guglielmo Monaco, Maximilian Tiffner, Antonia Di Mola, Wouter Herrebout, Mario Waser, Antonio Massa

**Affiliations:** 1 Dipartimento di Chimica e Biologia, Università di Salerno, Via Giovanni Paolo II, 132, 84084 Fisciano, SA, Italy; adimola@unisa.it; 2 Institute of Organic Chemistry, Johannes Kepler University Linz, Altenbergerstr. 69, 4040 Linz, Austria; maximilian.tiffner@gmail.com (M.T.); mario.waser@jku.at (M.W.); 3 Department of Chemistry, University of Antwerp, B-2020 Antwerp, Belgium; wouter.herrebout@uantwerpen.be

In this note, we report a correction to the published article, *Molecules* **2020**, *25*, 2272; doi:10.3390/molecules25102272 [1], regarding the absolute configuration (AC) of the used catalyst **IV** in the preparation of the sample sent for a VCD analysis.

In the paper, the VCD analysis was performed on the sample (+)-**1** to determine its absolute configuration and it was found that the AC is (*R*). This new compound was in fact synthetized with the catalyst (*S,S*)-**IV** (*ent*-**IV** in the article), while in the text it was erroneously reported that the used catalyst was **IV** with the (*R,R*) configuration. 

Therefore, the reaction scheme should be corrected as follows:

**Scheme 1:**
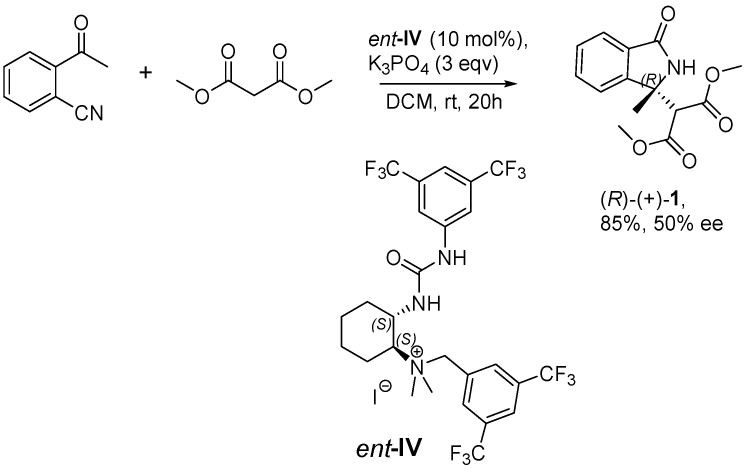
Asymmetric cascade reaction of 2-acetylbenzonitrile with dimethylmalonate.

Some other parts of the article must be corrected, as on page 4:

Under the best conditions of entry 1 of Table 3, the reaction was scaled up to 100 mg of 2-acetylbenzonitrile, using catalyst *ent*-**IV** (with *S*,*S* configuration), obtaining similar results in terms of yield and ee (90% and 50%, respectively). The enantiopurity of the product was further improved by means of a heterochiral crystallization process (**1** crystallizes as a racemate), leading to the isolation of **1** from the mother liquor in up to 96% ee and in an acceptable efficiency (45% yield), thus resulting in an overall process (asymmetric catalytic cyclization followed by crystallization) allowing for considerable quantities of almost enantiopure isoindolinone **1** from simple starting materials. This sample was used for the determination of the Absolute Configuration by VCD.

In addition, the proposed reported mechanism of Scheme 2 [1] must be revised considering different interactions in the TS, as follows:

The nature of this exact interaction mode remains speculative, but it is obvious that the bifunctional nature of the catalyst is crucial for obtaining the promising enantioselectivities achieved herein (the *S,S*-catalyst gives mainly the *R* product; the configuration is determined as described in the following chapter).

The experimental section, in particular:

*3.2. Procedure of Asymmetric Synthesis and Crystallization of***1**

In a typical procedure, a mixture of 2-acetylbenzonitrile (14 mg, 0.1 mmol) in CH2Cl2 (1.8 mL), catalyst **IV** (5 mol%), anhydrous K3PO4 (64 mg, 3 eqv.), and dimethylmalonate (34 μL, 3 eqv.) was stirred at room temperature until the disappearance of the starting material (TLC, Hexane/Ethyl acetate, 4:6). The solution was filtered and purified on silica gel (Hexane/Ethyl acetate, 70:30 to 50:50) obtaining a white solid. Yield: 85% (23.5 mg, 0.085 mmol). The spectroscopic data are in accordance with the literature [12]. Ee: 50%, Chiralpak IA3, Hex/IPA 80:20, 0.6 mL/min, λ: 254 nm, t: 17.52 min and 20.90 min. The reaction was also scaled up to 100 mg of 2-acetylbenzonitrile with cat. *ent*-**IV** with an (*S,S*) configuration (0.714 mmol) giving similar results in terms of yield and ee of the opposite enantiomer. Procedure for crystallization of **1a**. A sample of 40 mg, obtained from the above scale-up procedure, was dissolved in a mixture of CHCl3 (500 μL) and hexane (1.00 mL) at room temperature and then left at −20 °C for 72 h. The enantio-enriched product was recovered by filtration and evaporation of the solution, yielding 18 mg of (*R*)-**1** with 96% ee. [α]^20^*D*: +104.0 (c 0.80, CHCl_3_). This sample was then used in VCD analysis.

The authors state that the scientific conclusions are unaffected. The original publication has also been updated.

## Figures and Tables

**Scheme 2:**
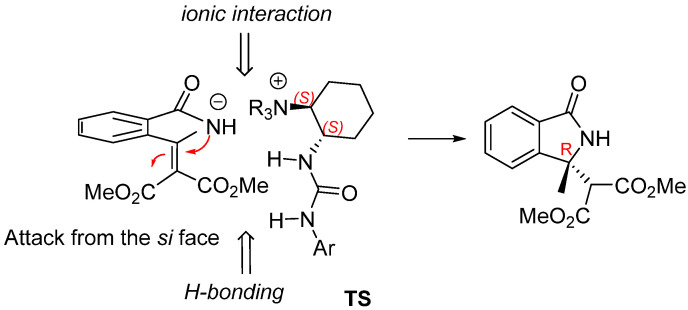
Proposed transition state.
