# Supplementary material for: Chiral Phase Transfer Catalysis in the Asymmetric Synthesis of a 3,3-Disubstituted Isoindolinone and Determination of Its Absolute Configuration by VCD Spectroscopy
Source: Molecules. 2020 May 12;25(10):2272. doi: 10.3390/molecules25102272 (PMC7287911; doi:10.3390/molecules25102272)
Supplement: Supplementary file 1 [file molecules-25-02272-s001.pdf]

Chiral phase transfer catalysis in the asymmetric synthesis of a 3,3-disubstituted isoindolinone and determination of its absolute configuration by VCD Spectroscopy

Guglielmo Monaco,* Maximilian Tiffner, Antonia di Mola, Wouter Herrebout, Mario Waser and Antonio Massa*

SUPPORTING INFORMATION

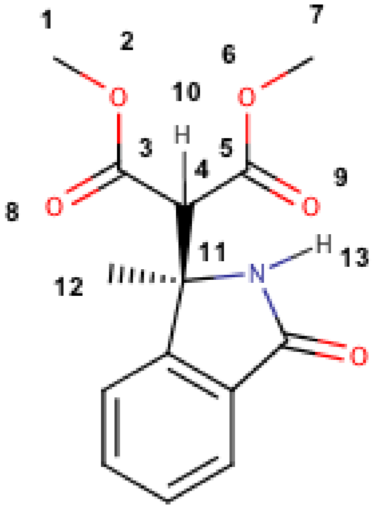

Figure S1. Atomic numbering used to define the geometrical parameters of the conformers of 1.

Table S1. Geometrical parameters of the conformers of **1.** Δ*E* in kcal mol^{-1}. With reference to the numbering of Figure SI-F1, the atoms defining dihedral angles and distances are in parentheses: $\vartheta 1$(1,2,3,4), $\vartheta 2$(2,3,4,5), $\vartheta 1$(1,2,3,4), $\vartheta 3$(3,4,5,6), $\vartheta 4$(4,5,6,7), $\vartheta 5$(10,4,11,12), φ (8,3,5,9), d1 (13,9), d2(13,8), d3(13,6) , d4(13,2)

Conf#	Δ*E*	$\vartheta 1$	$\vartheta 2$	$\vartheta 3$	$\vartheta 4$	$\vartheta 5$	d1	d2	d3	d4	φ
1	0.00	178	28	-104	179	-174	2.26	4.93	4.13	5.1	-69
2	0.56	-177	-49	-68	178	52	4.62	2.25	3.14	4.08	-176
3	0.88	177	136	-72	176	54	3.26	2.27	4.59	4.09	43
4	1.14	-179	63	37	180	169	2.28	5.21	3.91	4.66	159
5	1.72	-179	176	-90	176	-170	4.07	4.94	2.2	5.05	64
6	2.79	177	139	83	-176	65	3.06	4.2	4.33	2.28	-106
7	2.89	-180	52	-129	-177	-57	4.94	4.09	5.02	3	-55
8	2.93	-178	-39	92	-176	63	4.33	4.2	3.05	2.27	45
9	3.12	179	47	54	178	-54	4.93	3.03	5	4.02	158
10	3.55	178	-79	-16	-177	157	3.51	4.97	2.55	4.92	-134
11	3.61	179	47	61	-179	63	3.35	4.08	4.29	2.37	168
12	3.68	-177	178	74	-175	-167	4.14	5.11	2.23	4.89	-79
13	5.38	-180	131	75	180	-55	5.18	3.59	4.77	3.9	-119
14	6.13	-176	-161	-115	-179	-48	5.23	3.84	4.88	3.18	70

Table S2. Rotational strengths for the three carbonyl resonances obtained for the 14 conformers of **1**. R1, R2 and R3 are the rotational strengths in order of increasing frequency. ***φ*** is the dihedral angle O=C–C=O. Couplets have been assigned positive/negative when, of the two strongest dichroic signals, the one at lower frequency is positive/negative. When the third signal has an intensity higher than half the average absolute value of the two strongest dichroic signals, a star has been added, indicating a possible oversimplification by use of the two-carbonyl exciton chirality method.

#conf	***φ***	ECO			DFT			couplet_ECO	couplet_DFT
		R1	R2	R3	R1	R2	R3		
1	-69	-245	230	16	-447	-32	290	negative	negative
2	-176	-285	273	12	-427	338	48	negative	negative
3	43	222	272	-494	46	-416	399	positive	negative
4	159	90	65	-155	-130	400	-117	positive*	negative
5	64	434	3	-437	267	-142	-278	positive	positive*
6	-106	-321	51	271	49	-167	83	negative	negative
7	-55	-384	-94	478	-172	-393	341	negative	negative
8	45	192	-325	133	2	-99	-18	positive*	-
9	158	184	20	-204	27	106	-247	positive	positive
10	-134	-275	-40	315	-108	-207	275	negative	negative
11	168	280	-255	-25	214	-221	-73	positive	positive
12	-79	-424	-164	588	-147	-189	305	negative	negative *
13	-119	-10	-318	328	-147	230	-2	negative	negative
14	70	553	-416	-137	308	-174	-165	positive	positive*

HPLC trace for *rac*-**1** prepared according to ref. 1.
Conditions for separation: Chiralpak IA3, Hex/IPA 80:20, 0.6 mL/min, λ 254 nm

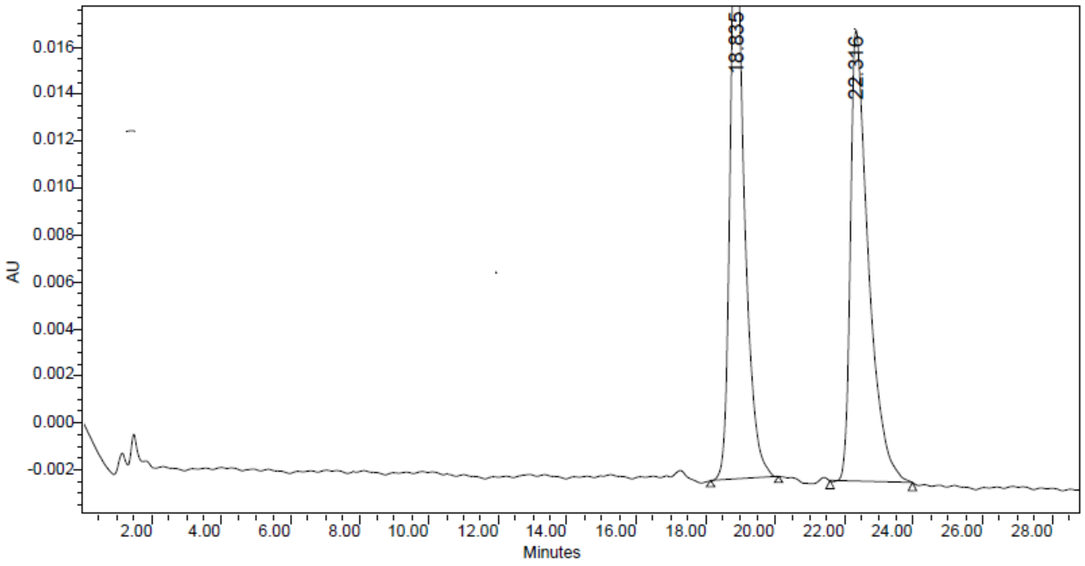

	RT (min)	Area (µV*sec)	% Area	Height (µV)	% Height
1	18.835	707402	50.35	25012	56.53
2	22.316	697636	49.65	19235	43.47

HPLC trace for enantioenriched *R*-**1** (experiment coming from scale-up).

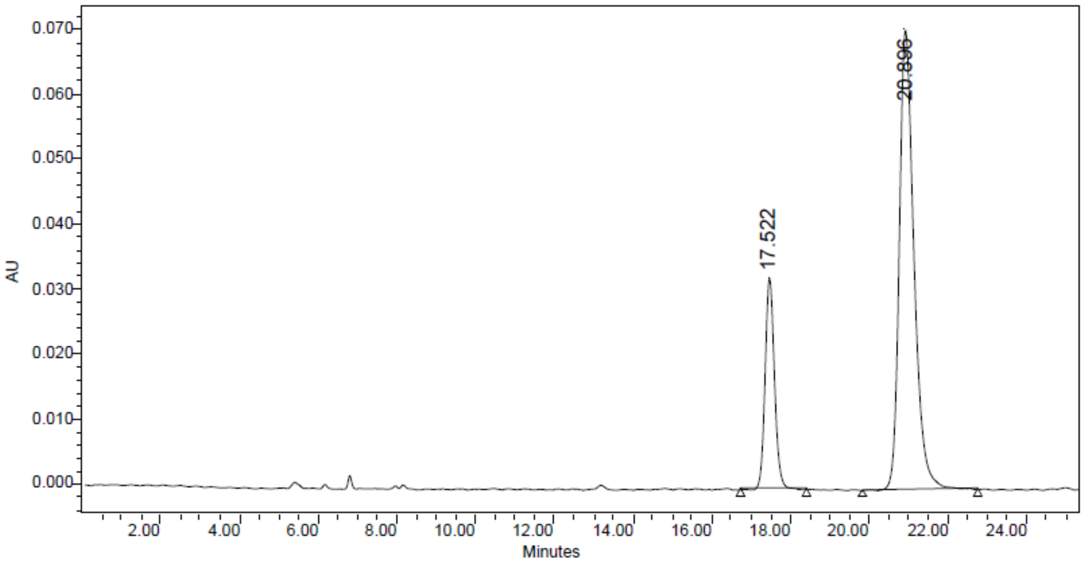

	RT (min)	Area (µV*sec)	% Area	Height (µV)	% Height
1	17.522	8323442	24.96	32519	31.48
2	20.896	25027896	75.04	70771	68.52

HPLC trace for enantioenriched *R*-**1** after crystallization

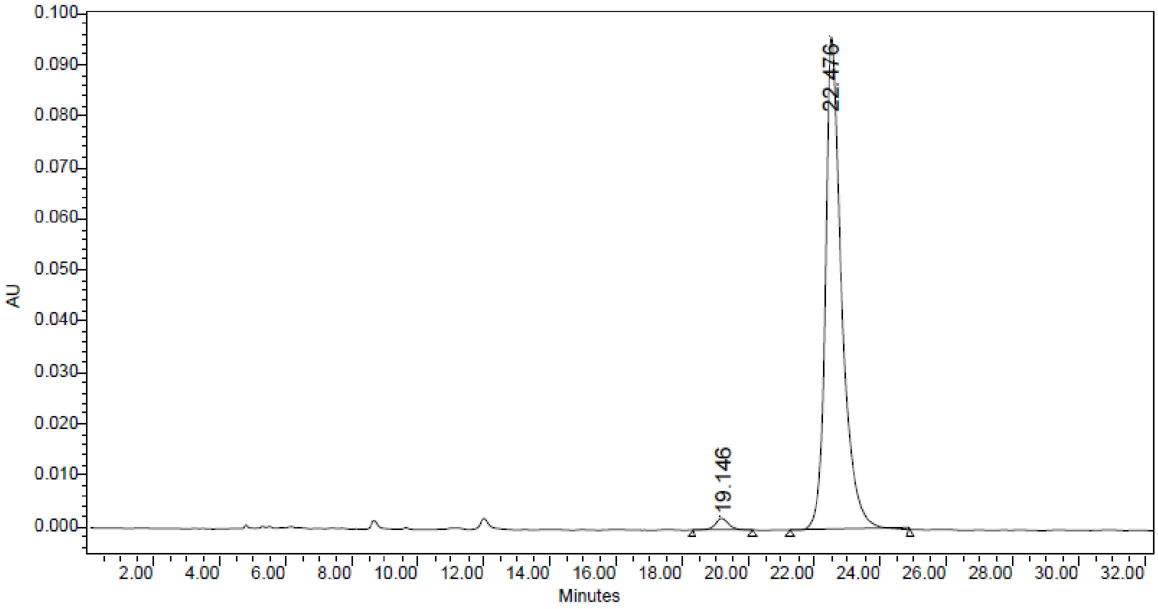

	RT (min)	Area (□V*sec)	% Area	Height (□V)	% Height
1	19.146	64493	1.92	2298	2.34
2	22.476	3301683	98.08	95865	97.66

Reference

1. Di Mola, A.; Di Martino, M.; Capaccio, V.; Pierri, G.; Palombi, L.; Tedesco, C.; Massa, A. Synthesis of 2-Acetylbenzonitriles and Their Reactivity in Tandem Reactions with Carbon and Hetero Nucleophiles: Easy Access to 3,3-Disubstituted Isoindolinones. *Eur. J. Org. Chem.* **2018**, 1699–1708.
